# Management of Intracranial Metastatic Disease With Laser Interstitial Thermal Therapy

**DOI:** 10.3389/fonc.2018.00499

**Published:** 2018-10-31

**Authors:** Afshin Salehi, Ashwin A. Kamath, Eric C. Leuthardt, Albert H. Kim

**Affiliations:** Department of Neurological Surgery, Washington University School of Medicine, St. Louis, MO, United States

**Keywords:** laser interstitial thermal therapy (LITT), intracranial metastatic disease, brain metastases, overall survival (OS), thermal-damage-threshold (TDT)

## Abstract

Treatment approaches for metastatic brain tumors continue to evolve, with increasing recent emphasis on focal therapies whenever possible. MRI-guided Laser Interstitial Thermal Therapy (LITT) is a minimally invasive surgical option that has broadened the capability of the neurosurgeon in treating difficult-to-treat intracranial lesions. This technology uses image-guided delivery of laser to the target lesion to generate heat and thereby ablate pathological tissue and has expanded the neurosurgical armamentarium for surgical treatment of brain metastases. In this study, we describe the indications for LITT in the management of intracranial metastatic disease and report our institutional experience with LITT.

## Introduction

Current strategies for the treatment of metastatic brain tumors include surgical resection or ablation, stereotactic radiosurgery, fractionated radiation therapy, whole brain radiation therapy (WBRT), and in select cases, targeted medical therapy. Recent data indicate that rather than performing WBRT, more focused and localized treatment of brain metastases using stereotactic radiosurgery (SRS) might be favorable due to cognitive issues associated with WBRT (1). These results also raise the general concept that focal therapies, where possible, should be preferentially considered for brain metastases. Additionally, due to advances in the treatment of systemic disease in this diverse group of patients, practitioners are encountering a growing number of patients with brain metastases (2) and particularly patients who fail first- and even second-line therapy for their intracranial disease.

Laser Interstitial Thermal Therapy (LITT) is a novel, highly focused, minimally invasive technique that can be used to treat a variety of solid organ tumors (3, 4). The development of complementary technologies, such as intraoperative magnetic resonance imaging (MRI) and real-time MRI thermometry has enabled LITT to enter the fields of neurosurgery and neuro-oncology (5–10). In recent years, LITT has been applied to intracranial lesions, including metastatic disease to the brain, and has yielded safe and satisfactory treatment results with significantly less morbidity (11) and shorter hospital stays than traditional open craniotomy (7).

Proper patient selection for the appropriate indication is of utmost importance in ensuring the success of LITT. Firstly, patients must be willing to undergo a surgical procedure and be able to medically tolerate general anesthesia. In general, the indications fall into the broad categories of LITT as salvage therapy or frontline therapy. LITT has been used as frontline therapy in surgically inaccessible tumors (12), such as thalamic or basal ganglia gliomas. Other work has shown LITT to be effective in managing metastases that fail radiosurgery (13, 14) and in radiation necrosis (13, 15). Conveniently, in cases where the diagnosis is uncertain or would affect subsequent management, LITT can be performed subsequent to stereotactic needle biopsy during the same procedure.

LITT uses an MRI-compatible optical probe that transmits laser light through to a sapphire tip. The probe is inserted into the brain lesion with stereotactic guidance via a stab incision and a simple burr hole. The laser then produces a controlled thermal injury to the surrounding tissues. MRI thermometry allows for continuous monitoring of ablation in a controlled manner. The LITT procedure is generally well-tolerated with low operative morbidity (11), which is especially desirable in the treatment of cancer patients who often have significant systemic burden of disease. LITT therefore offers a promising treatment modality for intracranial metastatic disease. In this study, we describe our institutional series of 25 cases of intracranial metastatic disease treated with LITT.

## Methods

### Study design

Institutional review board approval was obtained for this research (IRB #201609152). A retrospective database of LITT patients was maintained and included demographics, age, sex, indications for LITT, lesion type/location/dimensions as well as operative data, such as procedure time, number of trajectories, post-operative complications and readmission rates. Patients were followed post-operatively. Overall survival was determined as time from surgery until the time of death or time of last visit. PFS was measured from the time of surgery until evidence of tumor progression, time of last stable image, or death.

### Operative technique

The LITT procedure at our institution has previously been described in detail (8, 9). In brief, for all procedures, Stealth navigation (Medtronic Inc., Minneapolis, MN, USA) was used for stereotaxy and trajectory planning. A registration error of less than 2 mm was used as a general goal. Intra-operative MRI (IMRIS Inc, Minnetonka, MN, USA) was used for real-time MRI thermography of the treatment zone. The planned trajectory was evaluated in detail to avoid sulci and blood vessels, and generally, the trajectory chosen was in line with the long axis of the lesion. All patients received advanced MR imaging, including diffusion tensor imaging (DTI), which was used to avoid passage through eloquent white matter. Earlier in our series, we tended to use the Monteris Axiis® frame while later in the series, most cases were performed with the Monteris® Mini-Bolt as the laser base, which has a low profile (142 mm with driver). Yet in instances with superficial lesions, the Axiis® frame is advantageous due to less artifact superficially in the cranium compared to the Mini-Bolt. Typically, the stereotactic trajectory was aligned using the Vertek® arm (Medtronic Inc). A handheld Stryker drill was used to generate a 4.5 mm burr hole, through which the Monteris® bolt was screwed into the skull. Either diffusion tip or a side-fire tip was inserted stereotactically into the tumor. Next, the intra-operative MRI was brought into the operative theater, and initial imaging obtained to confirm probe placement. The surgeon then delivered laser therapy to the lesion while monitoring real-time thermography via MRI to achieve appropriate heat dose delivery. Post-operatively, patients were treated with Keppra and a 2-to-3-weeks taper of dexamethasone.

### Statistics

The Kaplan-Meier (KM) product limit method was used to estimate empirical survival probabilities, including overall survival and progression-free survival. Log-rank test was applied to compare survival between patient groups. KM curves were generated. Progression-free survival was determined as the time from surgery to recurrence, date of last stable scan, or death. Multivariate Cox proportional hazard model was applied to include multiple covariates for survival analysis. Hazard ratio (HR) with 95% confidence interval was calculated from Cox proportional hazard model.

## Results

### Participants

A total of 25 LITT cases were performed for metastatic brain tumors on 24 patients between September 2010 to April 2016 at Washington University in St. Louis (Table 1). There were 15 males and 9 females with an average age of 59 years (range 38–74). Tumor types ranged from primary origin of lung (*n* = 16), melanoma (*n* = 3), followed by breast, colon, ovarian, and unknown primary. The majority of the lesions were frontal (*n* = 11) followed by parietal (*n* = 8) and other locations (*n* = 6) (Table 1). The mean follow-up period was 16.05 months (range 0.7–46.73).

**Table 1:** This table shows the demographic of the overall population.

**Sex, *n* (%)**
Male	15 (62)
Female	9 (38)
Total	24
Average age, years	59 (38–74)
**Primary tumor type**, ***n*** **(%)**
Lung	16 (64)
Melanoma	3 (12)
Breast	2 (8)
Colon	1 (4)
Ovarian	1 (4)
Unknown	2 (8)
Total cases	25
**Location**, ***n*** **(%)**
**Lobar**	
Frontal	11 (44)
Parietal	8 (32)
Temporal	1 (4)
Occipital	1 (4)
Insular	1 (4)
**Deep**	
Thalamic	1 (4)
Basal Ganglia	1 (4)
Cerebellar	1 (4)
Total	25

*There were 24 patients who underwent 25 cases*.

### Indications and operative details

LITT was chosen as the first-line therapy in only two cases for which “difficult to resect” location was the primary indication. For the rest of the cases (*n* = 23), it was chosen as a secondary or salvage therapy. In this latter group, surgeons indicated location as the primary reason for LITT in five of the cases, failure of prior treatments as the primary reason in 13 cases, and old age as well as poor functional status in the remaining 5 cases. Six patients had craniotomy and radiation therapy performed prior to LITT. Four patients had previous craniotomy and SRS/Gamma knife. Six patients had prior SRS and no craniotomies.

The average lesion volume was 7.32 cm^3^ (range 1.00–24.59). Treatment areas were monitored via standard thermal dose threshold (TDT) lines, with yellow line signifying the thermal dose equivalent of 43°C for 2 min and the blue line 43°C for 10 min. TDT lines were not available for two of the cases. Of the remaining 23 cases, 12 (53%) and 6 (26%) achieved complete coverage of the contrast-enhancing lesion by the yellow and blue TDT lines, respectively, with average coverage in the overall cohort of 95% and 92% by the yellow and blue TDT lines. Complete coverage of the lesions was limited by ablation area encroaching on eloquent regions, presence of heat sinks, such as ventricles or blood vessels near the ablation area, or prohibitively large size of the lesions. In 5 (21%) cases, two trajectories were used to ablate tumor. Postoperative MRI was performed on postoperative day 1 for evaluation of the extent of ablation and establishment of a baseline.

Time of surgery was comparable to craniotomy with an average of 219 min (range 105–490). Although not statistically significant, there was a downward trend over time in operative time (*R*^2^ = 0.21, data not shown). There was no correlation between surgery time and tumor size or location (data not shown).

### Complications

There was one (4%) perioperative complication and 4 (16%) later complications leading to unplanned readmissions within 30 days (Table 2). The perioperative complication was a seizure that occurred in a patient with a large tumor (10.09 cm^3^). He was given a dose of 2 mg Ativan and 200 mg of Vimpat which abated the seizures and was discharged in stable condition to rehab on POD 6 on Keppra and Vimpat. One of the re-admission cases was also potentially due to seizures with presentation of altered mental status on POD 16. After treatment, this patient and family opted for comfort care. Two of the readmissions were due to edema, one of which was secondary to hyponatremia and responded well to correction of the sodium and the other, transient hemiparesis, which resolved with a course of steroids. Analysis of the patients who suffered a complication/readmission showed that cases with complications were associated with tumors with larger volume (mean volume 12.32 cm^3^ ± 7.4) compared to those who had no complications (mean volume 5.93 cm^3^ ± 4.96) (unpaired *t*-test, *p* = 0.032).

**Table 2:** Complication table.

**Patient**	**Pathology**	**Age, years**	**Sex**	**Volume, cm^3^**	**Type**	**POD**	**Complication**	**Management**
1	L Frontal Melanoma	60	M	10.09	Perioperative	1	Seizures	Antiepileptic
2	R Frontal Melanoma	56	M	8.91	Readmission	4	Confusion	Negative workup. Sent to rehab on day 2
3	R Parietal Breast	59	F	12.8	Readmission	8	Edema, Left-sided hemiparesis	Edema treated with steroid
4	L Parietal Lung	58	F	5.3	Readmission	10	Aphasia, edema, hyponatremia	Fluid restriction, hypertonic saline, rehab on day 3
5	R Parietal Lung	65	M	24.59	Readmission	16	AMS, seizures	Made comfort care^*^

*One perioperative complication and four readmissions*.

* *Patient 5 came back on post-operative day 16 with altered mental status believed to be secondary to seizures who opted for comfort care and ultimately expired. POD, post operative day; AMS, altered mental status*.

### Outcome data

**Figure 1:**
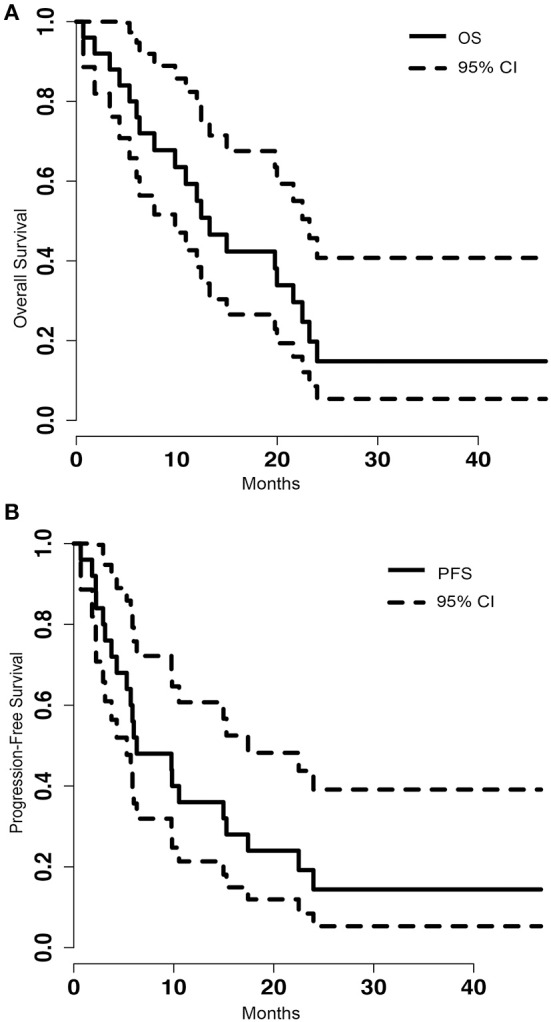
**(A)** Kaplan-Meier graph of the overall survival (OS) of the population and the 95% confidence interval (CI). The median OS was 13.27 (95% CI = 9.83–23.20). **(B)** Shows the progression free survival (PFS) of the population and the 95% CI. The median PFS was 6.30 (95% CI = 5.3–17.43).

Of the 25 metastatic brain tumor cases treated with LITT, tumor volumetric and blue TDT line coverage data were available on 23 patients. Eight of the cases (32%), either had biopsy performed at the time of LITT to confirm the diagnosis of metastasis or did not have prior SRS or RT. Zero patients were lost to follow-up. At the time of analysis, five (21%) patients were still alive, with a mean follow-up of 32.26 months (range 7.20–46.73). Among the 19 expired patients, we can identify systemic disease burden as the cause of death in four patients and CNS disease as cause of death in six patients whereas specific cause of death cannot be determined in the remaining nine patients. The median overall survival (OS) was 13.27 months [95% confidence interval (CI) = 9.83–23.20] (Figure 1A). The median progression-free survival (PFS) was 6.30 months (95% CI = 5.3–17.43) (Figure 1B). Stratified by location, frontal (8 of 24), parietal (5 of 24) or other (6 of 24), did not make a significant difference on OS (*p* = 0.429) or PFS (*p* = 0.364).

**Figure 2:**
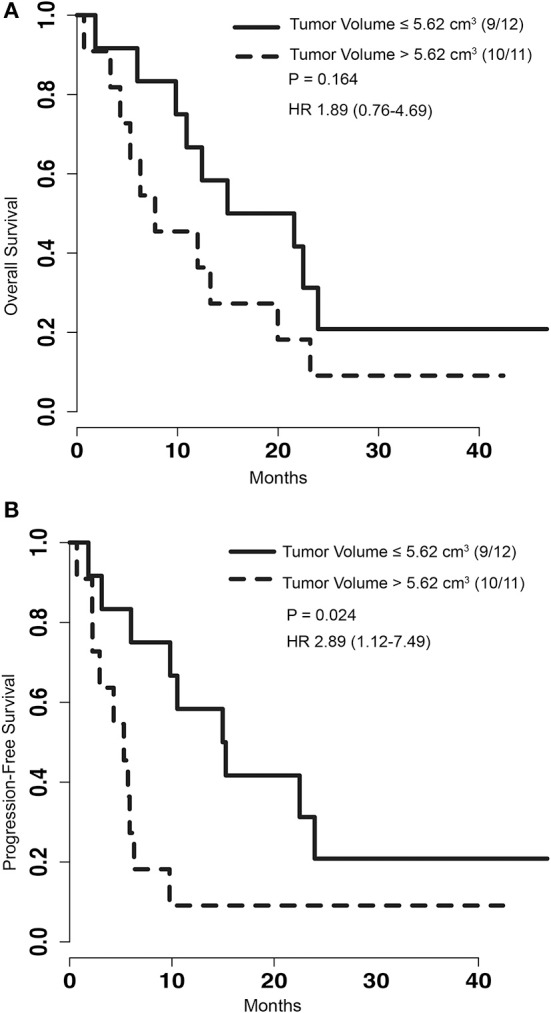
**(A)** Kaplan-Meier graph depicting the OS of patients with the group dichotomized based on the volume of tumors greater than 5.62 cm^3^ or less than and equal to 5.62 cm^3^. *P*-value for log rank test and hazard ratio are depicted on the graph. **(B)** Kaplan-Meier graph of PFS for patients with tumor volumes greater than 5.62 cm^3^ or less than and equal to 5.62 cm^3^. Log rank test comparing this two groups shows significantly improved survival for patients with smaller tumors (*p* = 0.024).

**Figure 3:**
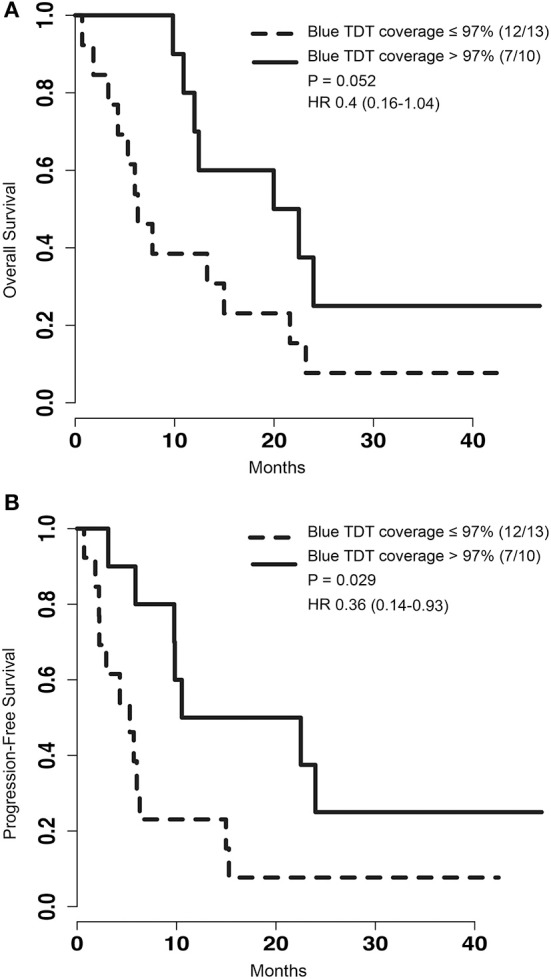
**(A)** Kaplan-Meier graph depicting the OS of patients with the group dichotomized based on the blue thermal dose threshold (TDT) coverage area of 97%. *P*-value for log rank test and hazard ratio are depicted on the graph. **(B)** Kaplan-Meier graph of PFS for patients with treatment coverage area greater than 97% or less than and equal to 97%. Log rank test comparing these two group shows a significantly improved survival for patients with blue TDT line coverage >97%.

To determine if pre-operative tumor size plays a role in outcome after LITT, pre-procedural tumor volumes were dichotomized with a cut-off at the median volume of 5.62 cm^3^. PFS of patients with tumor volumes greater than 5.62 cm^3^ was significantly shorter than that of patients with tumors smaller than or equal to 5.62 cm^3^ [*p* = 0.024, HR 2.89 (1.12–7.49)] (Figure 2B). However, analysis of OS between the same two groups did not show a significant difference [*p* = 0.164, HR 1.89 (0.76–4.69)] (Figure 2A). To determine if treatment coverage area based on the blue TDT line has an effect on outcomes, patients were dichotomized into two groups based on a treatment coverage cut-off at the median of 97%. PFS in cases with treatment coverage greater than 97% was significantly longer than those with less than or equal to 97% blue TDT coverage [*p* = 0.029, HR 0.36 (0.14–0.93)] (Figure 3B). OS of cases similarly dichotomized based on a 97% coverage area was not significantly different [*p* = 0.052, HR 0.4 (0.16–1.04)] (Figure 3A). Although dataset numbers are limited, an exploratory multivariate logistic regression analysis was performed to identify independent predictors of patient survival outcome. Four variants were included in the model—age, sex (M vs. F), percentage of blue TDT line coverage area (>0.97 vs. ≤ 0.97), and tumor volume (>5.62 vs. ≤ 5.62). Multivariate analysis did not show any significant association between any of the tested parameters and PFS or OS.

## Discussion

Our retrospective case series demonstrated that the PFS of patients with metastatic brain tumors treated with LITT is improved when greater than 97% of the tumor is treated to the blue TDT line (Figure 3). The OS of this group trended to significance (*p* = 0.052). This is consistent with similar studies describing that extent of surgical resection of metastatic tumors correlates positively with better outcome (16, 17). Lee et al. showed that median survival differs significantly when comparing gross total resection (median survival = 20.4) to subtotal resection (median survival = 15.1) (*p* = 0.016). Our findings suggest that even in cases of irregularly shaped tumors, use of single or multiple trajectories to achieve greater than 97% blue TDT treatment coverage, if safe, may be worth pursuing. A caveat of our study is the possibility that some of the lesions treated may have represented radiation necrosis. In our series, 32% of cases either had biopsy done at the time of LITT to confirm the diagnosis of metastasis or did not have prior SRS or RT, excluding the possibility of radiation necrosis at least in these cases. The lack of biopsy-proven tumor in the remaining cases is noted to be a limitation of our study. Nevertheless, recent studies have shown that LITT is also an effective treatment option for radiation necrosis in medically refractory cases (13, 15). The overall survival of 13.27 months seen in our group was comparable to that seen in other studies with surgery and radiation for recurrent metastatic cases. As another point of comparison, Koiso et al. retrospectively reviewed 859 patients with metastatic disease who underwent a second SRS and reported a median survival of 7.4 months (18).

Furthermore, we showed that larger pre-treatment tumor size is associated with worse outcome, with significantly shorter PFS and an increase in post-operative complications. However, as with our extent of TDT coverage data, we did not observe a clear impact of this factor on OS in patients. This can perhaps be explained by the fact that overall survival may be dominantly associated with systemic disease burden rather than central nervous system disease.

To keep complications at a minimum, patient selection is of great importance. LITT is ideal for lesions that are deeply seated and for which open surgery would be difficult, morbid, or at least transgress some amount of normal brain. However, LITT is also well-suited for more superficial lesions in patients who are too ill for surgery, have a thin scalp due to radiation or multiple prior surgeries, or have tenuous baseline functional status. Ideally, the target lesion for LITT would be (1) well-circumscribed such that the lesion could be treated within a 3 cm-diameter cylinder; (2) average to low vascularity; and (3) accessible via a safe linear trajectory that avoids inadvertent heating of eloquent structures. Additionally, the patient and laser apparatus combined must fit into the bore of the MRI scanner, which can be a limitation for obese patients. The efficacy of LITT as frontline therapy, particularly in small tumors, remains to be determined and will likely require a clinical trial testing the clinical benefit of LITT vs. SRS with a larger number of patients. But it is the opinion of the authors that safe supramarginal ablation by LITT might represent an interesting alternative to SRS.

With any surgical procedure, operative morbidity and different treatment options has to be weighed against possible benefits from surgery. In our series we had two cases (8%) of seizures (Table 2). As a comparison, Gokhale et al. showed risk of post-operative seizures after craniotomy to be about 7.3% with Keppra treatment (19), which is similar to the current study. Post-operative edema is another factor that must be considered with LITT. In our series, two cases experienced swelling requiring readmission. Post-LITT edema may potentially be more fulminant than post-craniotomy edema due to the lack of any decompression with LITT alone. A prolonged steroid course (2–3 weeks) or a minimally invasive craniotomy and limited resection immediately following LITT of larger tumors may represent strategies to mitigate this phenomenon (20, 21).

The management of incompletely treated tumors by LITT or recurrent tumors following LITT remains an open question. In four patients, LITT was repeated for recurrent lesions and in three cases the recurring tumors were treated with SRS. For larger lesions that may be incompletely ablated, there may be a role for adjuvant SRS for the residual. There may also be a role for administration of chemotherapeutic drugs in the post-LITT period given our prior finding that the BBB is permeable for 4–6 weeks post-LITT (22).

In summary, LITT is an increasingly attractive treatment modality for various types of intracranial lesions including brain metastasis. It offers a minimally invasive option for tumors that are difficult to access or refractory to prior treatment while at the same time offering comparable survival outcome to other salvage therapies.

## Author contributions

AS and AHK contributed to concept and design of the study. AS wrote the first draft of the paper as well as the major revisions. AHK edited drafts. AAK wrote sections of the manuscript. AHK and EL performed the surgeries and maintained the database. All authors contributed to manuscript revision, and have read and approved the submitted version.

### Conflict of interest statement

The authors declare that the research was conducted in the absence of any commercial or financial relationships that could be construed as a potential conflict of interest.
